# Doxorubicin-conjugated PLA-PEG-Folate based polymeric micelle for tumor-targeted delivery: Synthesis and *in vitro* evaluation

**DOI:** 10.1186/2008-2231-22-30

**Published:** 2014-03-06

**Authors:** Zahra Hami, Mohsen Amini, Mahmoud Ghazi-Khansari, Seyed Mehdi Rezayat, Kambiz Gilani

**Affiliations:** 1 Department of Medical Nanotechnology, School of Advanced Technologies in Medicine, Tehran University of Medical Sciences, Tehran, Iran; 2 Department of Medicinal Chemistry, Faculty of Pharmacy and Drug Design & Development Research Center, Tehran University of Medical Sciences, Tehran, Iran; 3 Department of Pharmacology, School of Medicine, Tehran University of Medical Sciences, Tehran, Iran; 4 Department of Toxicology & Pharmacology, Faculty of Pharmacy, Pharmaceutical Sciences Branch, Islamic Azad University (IAUPS), Tehran, Iran; 5 Aerosol Research Laboratory, Department of Pharmaceutics, School of Pharmacy, Tehran University of Medical Sciences, Tehran, Iran

**Keywords:** Doxorubicin, Folate, Micelle, PLA-PEG block copolymer

## Abstract

**Background:**

Selective delivery of anticancer agents to target areas in the body is desirable to minimize the side effects while maximizing the therapeutic efficacy. Anthracycline antibiotics such as doxorubicin (DOX) are widely used for treatment of a wide variety of solid tumors.

This study evaluated the potential of a polymeric micellar formulation of doxorubicin as a nanocarrier system for targeted therapy of a folate-receptor positive human ovarian cancer cell in line.

**Results:**

DOX-conjugated targeting and non-targeting micelles prepared by the dialysis method were about 188 and 182 nm in diameter, respectively and their critical micelle concentration was 9.55 μg/ml. The DOX-conjugated micelles exhibited a potent cytotoxicity against SKOV3 human ovarian cancer cells. Moreover, the targeting micelles showed higher cytotoxicity than that of non-targeting ones (IC_50_ = 4.65 μg/ml vs 13.51 μg/ml).

**Conclusion:**

The prepared micelle is expected to increase the efficacy of DOX against cancer cells and reduce its side effects.

## Background

Anthracycline antibiotics such as doxorubicin are widely used for treatment of a wide variety of solid tumors and hematological malignancies [1-3], but their clinical use is limited by their low water solubility, severe side effects such as cardiotoxicity and inherent drug resistance [4,5]. Drug delivery systems such as polymeric nanocarriers [6,7], liposomes [8] and dendrimers [9] can improve the antitumour efficacy and reduce toxicity of free DOX. Micelles that consist of hydrophilic shell and hydrophobic core are spherical nanoparticulate carriers with unique properties such as high solubility, high stability, appropriate size (20–200 nm) and long circulation in blood [10,11]. The use of polymeric micelles as carriers of anticancer drugs has been reported in previous studies [12-16].

Selective delivery of anticancer agents to target areas in the body is desirable to minimize the side effects while maximizing the therapeutic efficacy. Non-specific drug delivery often causes adverse effects on normal cells. Folate (FOL) has several advantages over various targeting ligands such as transferrin, peptides and antibodies. FOL has a small size, non-immunogenicity, low molecular weight and stability [17]. Therefore, micellar delivery systems have further been modified with target-specific ligands (FOL) to enhance tumor specificity and improve the tumor uptake by folate receptor-mediated endocytosis. Many approaches have also been described to prepare pH-sensitive micelles that can release the encapsulated drugs in acidic environment of tumors. Bae et al. have reported the pH-sensitive polymeric micelles based on poly (ethylene glycol)-poly(aspartate hydrazone-adriamycin) for DOX delivery that can release the drug in response to acidic pH at endosomes (pH 5.0–6.0) and lysosomes (pH 4.0–5.0). The micelles showed high antitumor activity in C-26 bearing mice [18]. Folate-poly(ethylene glycol)-poly (aspartate hydrazone-adriamycin) micelles For active intracellular drug delivery was also prepared. The micelles showed good antitumor effect in KB cell line [17]. In another study, Liua and co-workers prepared a targeting micelle based on poly(N-isopropylacrylamide-co-N,Ndimethylacrylamide-co-2-aminoethyl methacrylate)-b-poly (10-undecenoic acid) block copolymer and showed that the micelles was able to target the KB cells and release the drug in acidic pH of the tumor. The micelles significantly enhanced KB cell growth inhibition [19].

Covalent conjugation of anticancer drugs to their nanoparticulate carrier is more advantageous than physical encapsulation of drugs because it helps to stabilize the drug and prevent premature drug release into the blood circulation to assure drug delivery into the cancerous cells. Therefore, in this study, a folate functionalized PLA-PEG block copolymer was synthesized and doxorubicin was conjugated to the block copolymer via a pH-sensitive hydrazone bond. The prepared micelle was characterized for the structure of prepared block copolymer, average size and critical micelle concentration (CMC). The *in vitro* cytotoxicity of the folate targeting micelle against SKOV3 human ovarian cancer cells was evaluated using the 3-[4,5-dimethylthiazol-2-yl]-2,5-diphenyltetrazolium bromide (MTT) assay and compared with the folate-free micelle. Epithelial cancer cell lines such as SKOV3 demonstrate overexpression of folate receptors (FRs) [20]. MTT is a tetrazolium salt, which is reduced within the mitochondria in metabolically active viable cells. The resulting formazan crystals are impermeable to the cell membranes and accumulate only in uninjured cells [21], therefore this assay provides a measure of mitochondrial function following exposure to the test compound.

## Material and methods

### Instrumentation

The IR spectra were recorded on a Nicollet FT-IR Magna 550 spectrometer, Madison, USA. The ^1^H NMR spectrum was recorded on a Bruker DRX (Avance 500) spectrometer, Rheinstetten, Germany, 500 MHz. A double beam UV-Visible spectrophotometer (model 2100, Shimadzu, Japan) was utilized for spectrophotometric measurements. Dynamic light scattering (DLS) (Zetasizer Nano-ZS, Malvern Instruments Ltd., UK) was used to determine the dynamic diameter, size distribution and zeta potential of the micelles.

### Materials

Doxorubicin was purchased from RPG Life Sciences limited (Mumbai, India). L-lactide, poly(ethylene glycol) (PEG) with MW 4000 g/mol, Hydrazine, N-hydroxysuccinimide (NHS), dicyclohexylcarbodiimde (DCC), 1-ethyl-3-(3-dimethylaminopropyl) carbodiimide (EDC ), stannous octoate, folate and MTT were obtained from Sigma (St Louis, MO, USA). *p*-Nitrophenyl chloroformate (p-NPC), acetonitrile, toluene, dichloromethane and acetone (analytical grade) were purchased from Merck (Darmstadt, Germany). RPMI 1640 medium and penicillin/streptomycin solution were obtained from Gibco Invitrogen (Carlsbad, CA, USA). All other chemicals were of analytical grade.

### Preparation of folate-conjugated PLA-PEG block copolymer by ring opening polymerization

Preparation of PLA-PEG block copolymer containing terminal carboxylate group was started with the synthesis of monocarboxylated PEG according to the method described by WH Jo et al. [22] with some modifications and followed by ring-opening polymerization of the lactide in the presence of carboxylated PEG [23]. Briefly, vacuum-dried lactide (16 g) and carboxylated PEG (3 g) were allowed to react in anhydrous toluene in the presence of and tin (II) 2-ethylhexanoate (200 mg) as a catalyst at the refluxing temperature of toluene. The PLA-PEG copolymer was extracted by chloroform after evaporation of the reaction solvent. The prepared PLA-PEG–COOH (1 g) was activated by adding EDC (50 mg) and NHS (40 mg) in dimethyl sulfoxide (DMSO, 10 ml) 5 h at room temperature. To prepare the folate-functionalized copolymer, folate-NH_2_ was synthesized from reaction of folic acid (250 mg) and triethylamine (TEA, 0.5 ml) in the presence of NHS and EDC in methanol. After 2 h, 1 ml of ethylene diamine was added and the reaction continued overnight at room temperature. The prepared folate-NH_2_ was added to the activated copolymer in DMSO and the reaction continued for additional 48 h. The mixture was then dialyzed against deionized water to remove unreacted folate-NH_2_. The formation of monocarboxylated PEG and PLA-PEG copolymer was confirmed by infrared (IR) spectroscopy. The conjugation of folate to the copolymer was also confirmed by ^1^H NMR spectroscopy. The total amount of folate conjugated to copolymer was determined by UV spectroscopy at 365 nm.

### Preparation of doxorubicin-conjugated PLA-PEG block copolymer and micelle formation

Conjugation of Hydrazone Derivative of doxorubicin (Hyd-DOX) to the activated PLA-PEG-FOL block copolymer was carried out according to previously described method with some modifications [14]. The terminal PLA in copolymer was activated by adding *p*-NPC (340 mg) and dry pyridine (230 mg) to PLA-PEG-FOL (3 g) in dry methylene chloride at 0°C, followed by reaction at room temperature under nitrogen atmosphere. The conjugation of DOX-Hyd to freeze-dried activated block copolymer was performed in dry tetrahydrofurane under nitrogen atmosphere for 36 h. In the final step, dialysis method was employed to prepare the micelles. The folate-free micelles were also prepared in a similar way. The doxorubicin content in the copolymer was measured by UV spectroscopy at 490 nm.

### Particle size and zeta potential measurements by dynamic light scattering (DLS)

Particle size, polydispersity index (PDI) and zeta potential of blank and DOX-conjugated polymeric micelles were measured using a Zetasizer (Nano-ZS, Malvern Instruments Ltd., UK). All measurements were performed in triplicate.

### Determination of the critical micelle concentration (CMC)

The CMC of prepared micelles was estimated by fluorescence spectroscopy using pyrene as a fluorescent probe [24]. Briefly, 10 ml of DOX-conjugated copolymer aqueous solutions with different concentrations (0.05 μg/ml to 500 μg/ml) were added to the volumetric flasks containing solvent-dried pyrene (10^-7^ M). The solutions were sonicated at 40°C for 30 minutes, followed by stirring for 24 h at room temperature. The excitation wavelength was set at 334 nm and the intensity ratios of I_383_ to I_372_ were plotted as a function of concentration of the block copolymer solutions. The CMC of DOX-conjugated micelle was taken from the copolymer concentration at which the relative fluorescence intensity ratio began to increase.

### *In vitro* cytotoxicity studies

SKOV3 human ovarian cancer cells were cultured in RPMI-1640 medium supplemented with 10% fetal bovine serum (FBS) and 1% penicillin-streptomycin at 37°C in a humidified incubator with 5% CO2. The cytotoxic activity of free DOX and chemically conjugated DOX in targeting (DOX-Hyd-PLA-PEG-FOL) and non-targeting micelles against SKOV3 cells was measured using MTT assay. The cells were seeded in 96-well plates at 10,000 cells per well and incubated for 24 h before test. Free DOX and DOX-conjugated targeting and non-targeting PLA-PEG micelles were dissolved and diluted in the growth medium to give different concentrations of DOX (equivalent DOX concentrations; 10^-2^ - 10^5^ ng/ml). The blank micelle concentrations (0.01-100 μg/ml) in RPMI 1640 were also prepared. The old media in the plates were replaced with 100 μl of the media containing the test compounds. After 72 h incubation, 20 μl of MTT solution (5 mg/ml) was added to each well and the plates were then maintained in incubator for an additional 4 h. The media containing MTT were then removed and the purple formazan crystals were dissolved in 100 μl of DMSO. The absorbance of the formazan crystals was read with a Synergy HT Microplate Reader (Bio-Tek Instruments, Winooski, VT) at 570 nm. The IC50 values (The concentration of the test compounds at which 50% cell growth is inhibited) were calculated by using GraphPad Prism Software (Version 5.04, GraphPad Software, San Diego California, USA).

### Data analysis

All experiments were carried out three times and results were expressed as mean ± SD. All data analyses were performed using GraphPad Prism version 5.04. The significance level was set at p < 0.05.

## Results and discussion

### Preparation of doxorubicin-conjugated PLA-PEG-Folate micelle

**Figure 1:**
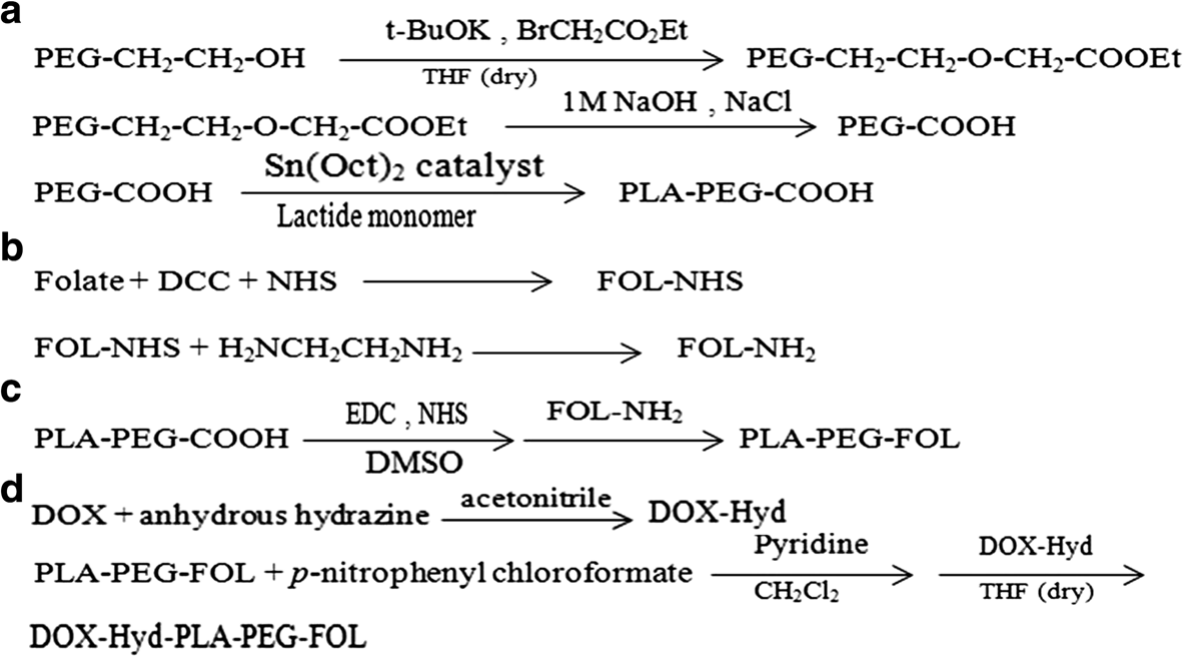
**Synthesis scheme of DOX-conjugated PLA-PEG diblock copolymer.** Synthesis of PLA-PEG-COOH **(a)**, Folate-NH_2_**(b)**, PLA-PEG-FOL **(c)** and DOX-PLA-PEG-FOL **(d)**.

**Figure 2:**
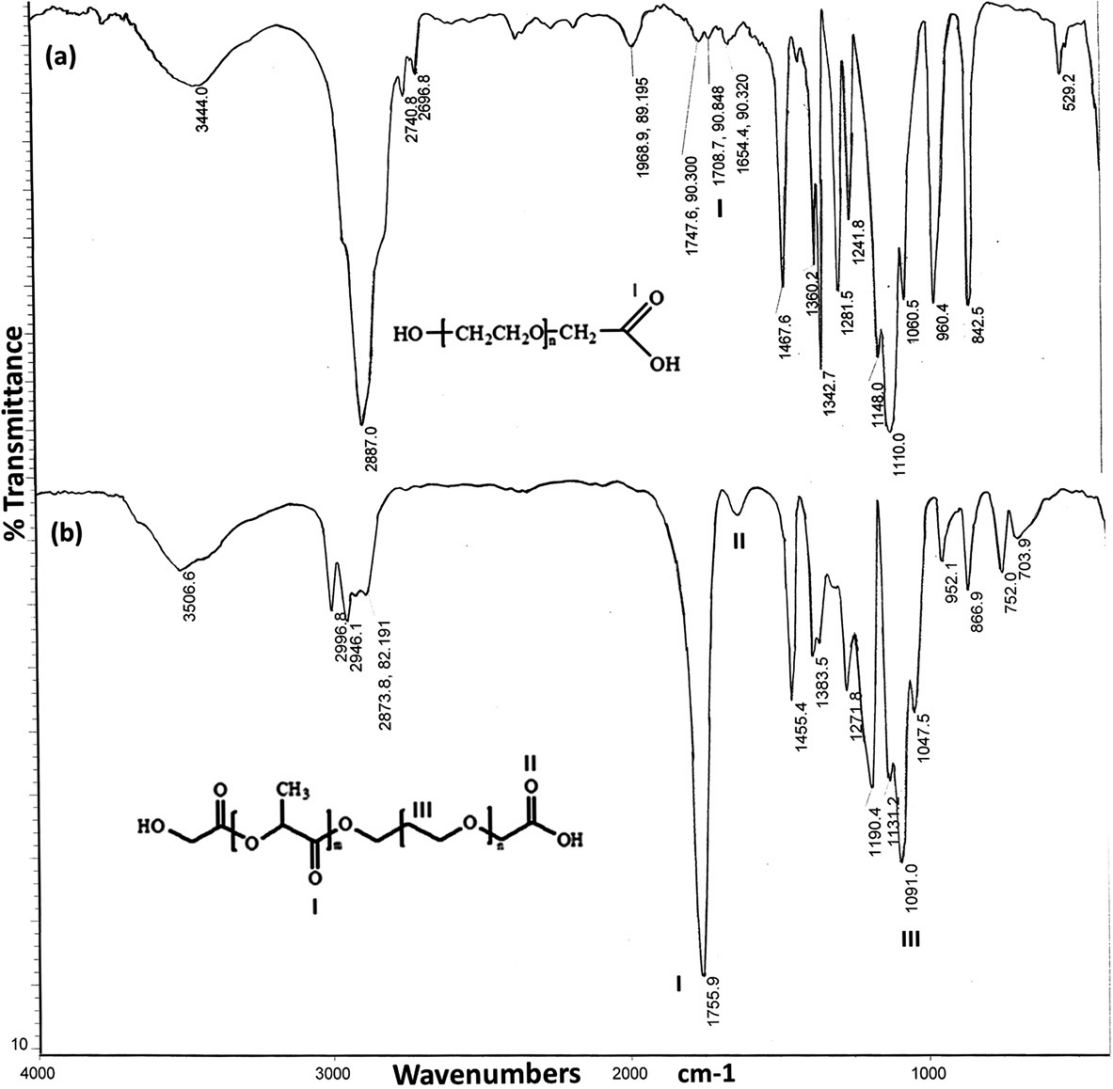
FTIR spectra of monocarboxylated PEG (a) and PLA-PEG-COOH conjugate (b).

**Figure 3:**
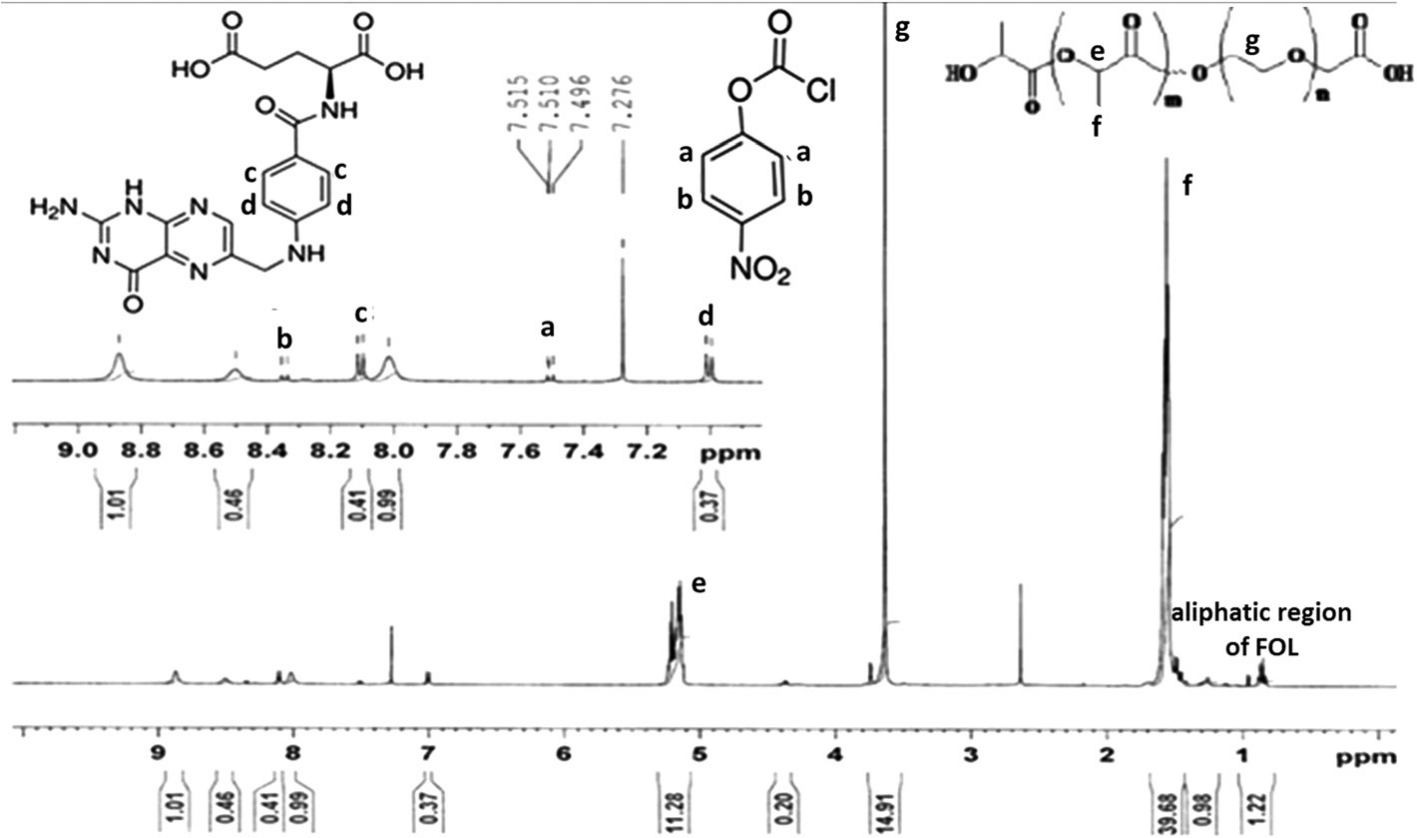
^**1**^**H NMR spectrum of p-nitrophenyl chloroformate-PLA-PEG-FOL (500 MHz, CDCl**_**3**_**).**

The synthesis pathway of DOX-Hyd-PLA-PEG-FOL is illustrated in Figure 1. PEG with two different end groups was first synthesized for the conjugation of folic acid to the carboxylate terminal group. The polymerization of lactide was then carried out in the presence of monocarboxylated PEG which PLA was conjugated to the hydroxyl terminal group of PEG. Spectral data of the prepared compounds are shown in Table 1. The structure of monocarboxylated PEG and PLA-PEG copolymer were confirmed by the IR spectrum (Figure 2). The peak at 1708 cm^-1^ in Figure 2(a) indicates the presence of carbonyl group of -COOH in the structure of carboxylated PEG. In the IR spectrum of PLA-PEG copolymer (Figure 2(b)), the characteristic sharp peak at 1755 cm^-1^ is attributed to the carbonyl (C = O) group in the PLA block of PLA-PEG-COOH. Besides, the peaks related to the C-O-C stretching and C = O of COOH in PEG appeared at 1091 and 1635 cm^-1^, respectively. The IR spectrum strongly indicates the ring opening polymerization of lactide and formation of PLA-PEG block copolymer. The^1^H-NMR spectrum (Figure 3) of prepared PLA-PEG copolymer was used to estimate the average molecular weight of the copolymer by comparing the peak ratio of the methylene protons of PEG (-OCH_2_CH_2_–: δ 3.6 ppm) with the peak ratio of methine protons of PLA (CH:δ 5.2 ppm). The molecular weight of PEG was 4000 g/mol, therefore the molecular weight of PLA-PEG copolymer was approximately 30,000. From these results, the degree of polymerization (DP) of lactate, n, is 373. In the next step, folate-NH_2_ was synthesized, as shown in Figure 1 and its conjugation to carboxylate-terminal group of PEG was carried out after activation of block copolymer. The effect of reaction solvent and temperature was evaluated in the synthesis of folate-NH_2_ and the best results were achieved at room temperature in methanol solvent. The conjugation of folate to the copolymer was confirmed by ^1^H NMR spectroscopy. The ^1^H NMR spectrum (Figure 3) shows the corresponding peaks of folate (small peaks at 7.1 ppm, 8.1 ppm and approximately 1 ppm) and *p*-nitrophenyl chloroformate (small peaks at 7.5 ppm and 8.4 ppm). The molar percent of folate in the copolymer is 66.8% measured by UV absorbance at 365 nm. The spectrum also shows the characteristic peaks of PEG (CH_2_: 3.6 ppm) and PLA (CH_3_: 1.6 ppm and CH: 5.2 ppm). To provide controlled release of doxorubicin, hydrazine was used as an acid-sensitive linker for DOX conjugation to the PLA backbone. Compared to the published method by Etrych et al. [25], the conjugation method was carried out in mild reaction conditions with a less time consuming and less costly reaction. The drug-conjugated micelle was prepared using dialysis method. The amount of DOX attached onto the copolymer was 39.6% (molar percent) determined by UV absorbance at 480 nm.

**Table 1:** Spectral data of the prepared compounds

**Compounds**	**IR (KBr) Vmax in cm**^**-1**^	^**1**^**H-NMR (CDCl**_**3**_**) δ in ppm**
PEG-COOH	1708 (C=O)	
PLA-PEG-COOH	1755 (C=O in PLA), 1091(C-O-C in PEG), 1635 (C=O in PEG)	3.6 (-OCH_2_CH_2_- in PEG), 1.6 (CH_3_ in PLA), 5.2 (CH in PLA)
*p*-NPC-PLA-PEG-FOL		7.1, 8.1, 1(FOL),
		7.5, 8.4 (*p*-NPC)

### Particle size distribution

The average diameter and zeta potential data of the blank, DOX-conjugated targeting and non-targeting polymeric micelles in deionized water measured using DLS technique are listed in Table 2. The mean diameters of the drug-free PLA-PEG-FOL micelles and the drug-conjugated targeting and non-targeting micelles were 176.71, 188.43 and 182.19 nm, respectively. In addition, the polydispersity index values indicate that the formed micelles had narrow size distribution. The particle size values were comparable to those obtained for other block copolymeric micelles with similar molecular weights [26]. In general, the size of nanoparticles affects their *in vivo* bio distribution. The size cut-off of tumor vasculature is about 200–700 nm [27]. The average size of both blank and DOX-conjugated micelles was smaller than 200 nm; therefore they are acceptable for drug delivery in cancer treatment. As presented in Table 2, the zeta potential values of micelles with folate ligand are more negative than that of non-targeting micelles which is due to the negative charged carboxyl groups of the folate ligand [28]. The negative charge enhances the dispersion stability of the micelles. Since the particle size and surface chemistry determine the fate of the micelles in blood circulation, the polymeric micelles prepared in this study may be appropriate for *in vivo* applications in cancer therapy.

**Table 2:** Particle size, polydispersity index and zeta potential of blank and DOX-conjugated micelles prepared by the dialysis method

**Zeta potential (mV)**	**Polydispersity**	**Particle size (nm)**	**Micelles**
-12.47 ± 2.92	0.25 ± 0.06	176.71 ± 12.61	Blank micelles
-10.24 ± 1.57	0.28 ± 0.04	188.43 ± 8.96	DOX-Hyd-PLA-PEG-FOL micelles
-7.12 ± 1.23	0.16 ± 0.05	182.19 ± 6.38	DOX-Hyd-PLA-PEG micelles

Values are presented as mean ± SD (n = 3).

### The CMC of DOX-conjugated PLA-PEG block copolymer

**Figure 4:**
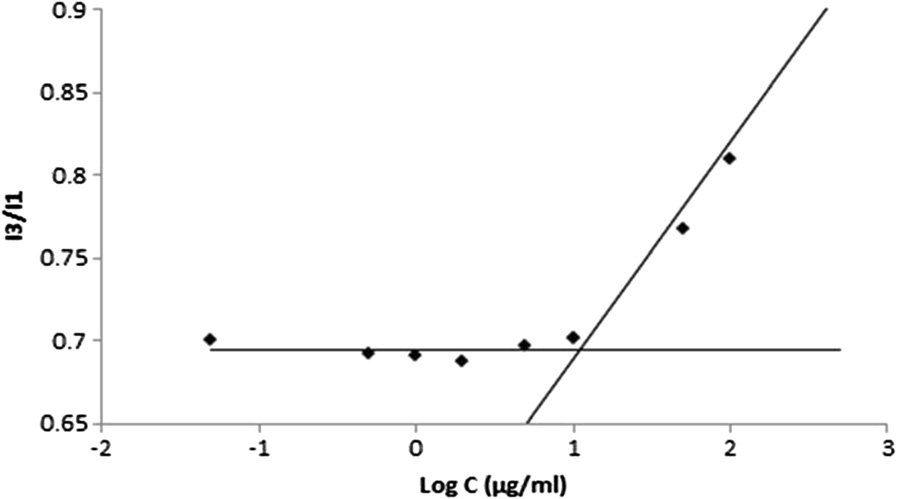
**The intensity ratio (I**_**383**_**/I**_**372**_**) of pyrene fluorescence as a function of DOX-Hyd-PLA-PEG-FOL concentration.**

The formation of micelles was studied using the CMC of DOX-Hyd-PLA-PEG-FOL determined by a fluorescence spectrophotometer measurement in the presence of pyrene as a fluorescent probe. The intensity ratio of the third band to the first band of the pyrene emission spectra (I_383_/I_372_) was used to evaluate the polarity of the pyrene environment [29]. The CMC of DOX-Hyd-PLA-PEG-FOL was 9.55 μg/ml as derived from Figure 4. Depending on the copolymer length, the CMC values of PLA-PEG copolymer reported in similar studies are 1–100 μg/ml [30,31]. The low CMC value reported in our study indicates that pyrene is located in a hydrophobic environment which is due to the relatively high molecular weight of the PLA backbone in the copolymer. Micelles with low CMC value exhibit a relative stability upon dilution *in vivo*[32], which is true in the case of prepared DOX-Hyd-PLA-PEG-FOL micelles.

### *In vitro* cytotoxicity studies

**Figure 5:**
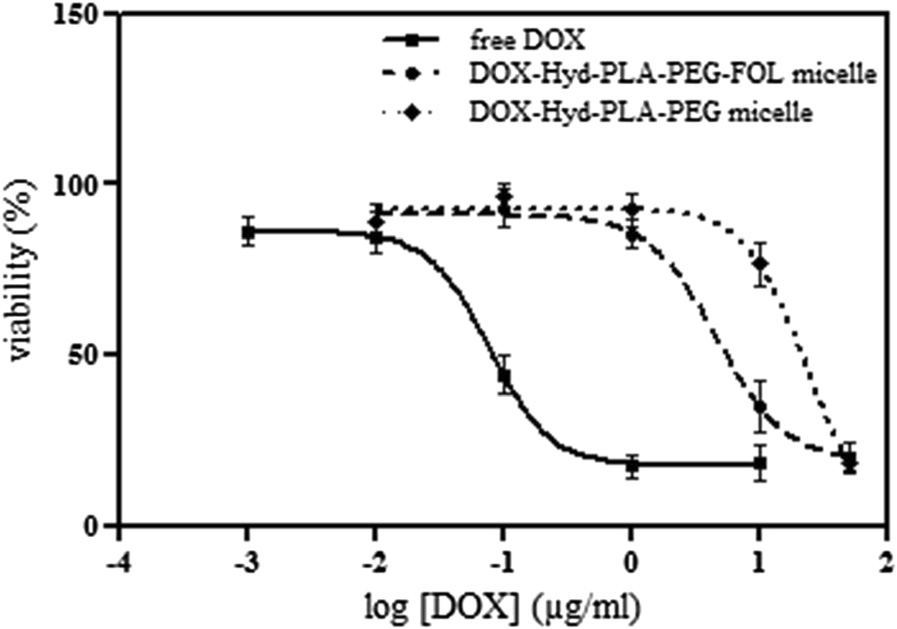
***In vitro *** **cytotoxicity of free DOX and DOX-conjugated targeting/non-targeting micelles against SKOV3 cell line after 72 h measured with the MTT assay. Cell viability is expressed as mean ± S.D.**

Figure 5 compared *in vitro* cytotoxicity of free DOX, DOX-Hyd-PLA-PEG-FOL micelles and DOX-Hyd-PLA-PEG micelles against SKOV3 cells as determined by the MTT assay. All three test compounds decreased the viability of the cells in a dose-dependent manner. The IC_50_ value of DOX, the concentration of DOX at which 50% cell growth is inhibited, was 0.08, 4.65 and 13.51 μg/ml for free DOX, DOX-conjugated targeting and non-targeting micelles, respectively. DOX-conjugated targeting micelles exhibited higher cytotoxicity than that of non-targeting ones (IC_50_ = 4.65 μg/ml vs 13.51 μg/ml). The overexpression of FRs on the surface of SKOV3 cells [33] enhanced the uptake of the DOX-Hyd-PLA-PEG-FOL micelles via FR-mediated endocytosis and resulted in 3-fold higher cytotoxicity. Free DOX also showed higher cytotoxic activity compared to DOX-conjugated targeting and non-targeting micelles. The higher cytotoxicity of free DOX seems to be due to the rapid diffusion of this small molecule into the cells, while the DOX-conjugated micelles are internalized into the cells via the endocytosis process. Moreover, the prepared micelles released DOX in a sustained and controlled manner using the acid labile hydrazone linkage (data not shown). Some other nanoparticulate delivery systems with sustained drug release also exhibited a less cytotoxic activity compared to the corresponding free drug [34-37]. It should be noted that the blank micelles did not show any cytotoxicity against SKOV3 cells (data not shown). The DOX-conjugated targeting micelles exhibited a potent cytotoxicity against SKOV3 cells, therefore the DOX-Hyd-PLA-PEG-FOL micelles can provide an effective treatment for ovarian cancer.

## Conclusions

Doxorubicin-conjugated PLA-PEG-folate micelles with the hydrazone linkage were prepared with active targeting capability. This formulation showed a superior cytotoxicity compared to non-targeting ones against a folate-receptor positive cell line. The prepared DOX-conjugated micelles with folate ligand, appropriate size and low CMC value have a great potential for *in vivo* applications in cancer therapy.

## Competing interests

The authors declare that they have no competing interests.

## Authors’ contributions

ZH: Carried out synthesis studies of block copolymer, micelle preparation and characterization, *In vitro* cytotoxicity studies and drafted the manuscript. MA: Supervisor and participated in polymer synthesis studies and carried out the interpretation of the NMR data. MGK: Supervisor and participated in design and interpretation of *in vitro* cytotoxicity studies. SMR: Supervisor and participated in the design of the study. KG: Supervisor, conceived of the study, and participated in its design and coordination and helped to draft the manuscript. All authors read and approved the final manuscript.
